# The Effects of Cell Phone Conversations on the Attention and Memory of Bystanders

**DOI:** 10.1371/journal.pone.0058579

**Published:** 2013-03-13

**Authors:** Veronica V. Galván, Rosa S. Vessal, Matthew T. Golley

**Affiliations:** 1 Department of Psychological Sciences, College of Arts and Sciences, University of San Diego, San Diego, California, United States of America; 2 Department of Liberal Arts, D’Youville College, Buffalo, New York, United States of America; Goldsmiths, University of London, United Kingdom

## Abstract

The pervasive use of cell phones impacts many people–both cell phone users and bystanders exposed to conversations. This study examined the effects of overhearing a one-sided (cell phone) conversation versus a two-sided conversation on attention and memory. In our realistic design, participants were led to believe they were participating in a study examining the relationship between anagrams and reading comprehension. While the participant was completing an anagram task, the researcher left the room and participants overheard a scripted conversation, either two confederates talking with each other or one confederate talking on a cell phone. Upon the researcher’s return, the participant took a recognition memory task with words from the conversation, and completed a questionnaire measuring the distracting nature of the conversation. Participants who overheard the one-sided conversation rated the conversation as significantly higher in distractibility than those who overheard the two-sided conversation. Also, participants in the one-sided condition scored higher on the recognition task. In particular they were more confident and accurate in their responses to words from the conversation than participants in the two-sided condition. However, participants’ scores on the anagram task were not significantly different between conditions. As in real world situations, individual participants could pay varying amounts of attention to the conversation since they were not explicitly instructed to ignore it. Even though the conversation was irrelevant to the anagram task and contained less words and noise, one-sided conversations still impacted participants’ self-reported distractibility and memory, thus showing people are more attentive to cell phone conversations than two-sided conversations. Cell phone conversations may be a common source of distraction causing negative consequences in workplace environments and other public places.

## Introduction

People spent an estimated 2.30 *trillion* minutes using their wireless devices over the last year [1]. Cell phones, one subset of these devices, are relied upon heavily for personal communication, and are often used in public spaces [2]. Recent surveys indicate that people are becoming more dependent on their cell phones. Cell phones are increasingly replacing land lines and participants described experiencing “a personal connection towards their cell phone” [3]. People have reported feeling so emotionally attached to their cell phones that they feel anxiety without their phones [4] or feel they “can’t live without” them [5]. With more technological capabilities, such as personal organizers and navigation devices, people see the cell phone as an essential part of their everyday lives and even feel that they have a “personal relationship” with their phones [6]. Seventy-six percent of respondents also said their cell phones were always on or on most of the time, and 24% felt they had to answer their cell phones “even when it interrupts a meeting or meal” [5]. With the increased presence and personal use of cell phones, there is a greater likelihood that an individual will be frequently impacted by cell phones because either they or someone around them is using a cell phone.

Most cell phone research has focused on how using a cell phone impacts the user. Much of the research has used a simulated driving task to examine these effects and has found that drivers using cell phones are slower to change speed or direction [7], and are slower to brake and miss more red lights, regardless of whether the device is held or “hands free” [8]. These cell phone effects are strong enough that the chances of being in an accident are similar to the risks of drunk driving [9], [10] and the effects are not likely to lessen with practice [11]. It is important to note that deficits appear to be related to cognitive, and not motor, effects since holding and dialing the phone were not significant factors [8]. Instead, it appears that impairment of visual attention leads to cognitive impairments such that drivers ‘see’ less [12] and remember fewer objects they directly looked at while driving [13].

While most research on cell phones has focused on drivers, more recent research has shown pedestrians are also affected by their cell phone usage. Pedestrians talking on cell phones have impaired visual attention while crossing the street [14], [15], [16] to such an extent that most pedestrians on cell phones did not see a clown riding on a unicycle nearby [17].

Cell phone use can clearly have a negative impact on the user’s cognition particularly when the user is driving or crossing streets on foot. A question worth considering is whether there is any impact on bystanders who overhear cell phone conversations, since there are possibly millions of bystanders to the millions of cell phone conversations that take place yearly. Surveys have shown that 82% of respondents are annoyed by others’ cell phone use in public [5] and that the level of annoyance depends on the public location [18]. However, only a few studies have experimentally addressed the effects of cell phone conversations that are overheard by bystanders. Monk and colleagues questioned bystanders who overheard either a one-sided or two-sided conversation at a bus stop or on a train [19], [20]. Participants found one-sided conversations more noticeable and intrusive. This difference was not due to the presence of a cell phone since both versions of the one-sided conversation (a person using a cell phone or a person who spoke in a normal tone with a partner who whispered) were viewed unfavorably [20]. Unfortunately, Monk and colleagues were limited by the transportation authorities to only five questions, and thus were unable to examine other issues about the different types of conversations.

The only study thus far to examine the cognitive effects of one-sided conversations on bystanders is a study by Emberson, Lupyan, Goldstein, and Spivey [21]. In this well-controlled study, participants were asked to ignore sixty seconds of speech that occurred while they completed verbal tasks and motor tasks. Participants were told they would hear speech from the computer speakers and were asked to focus their attention on the computer tasks. In the within-subject design, participants overheard different types of auditory distractions (one-sided, two-sided, monologue, and silence) over thirty-two verbal and motor trials. When compared to the silent baseline, participants were more distracted by overhearing a one-sided conversation than hearing either a monologue or two-sided conversations. In a follow-up study, Emberson and colleagues filtered the conversations, making the speech incomprehensible. Unlike their first study, they found no significant differences when comparing the monologue, two-sided and one-sided conversations to the silent condition. The researchers concluded cell phone conversations are more distracting than a typical dialogue because the content of a cell phone conversation is less predictable [21].

Our study tests the cognitive effects of one-sided conversation and serves as a bridge between research done in a well-controlled laboratory setting and a completely naturalistic setting. We were able, with the help of confederates, to have a realistic situation in a controlled environment. In contrast to Emberson et al. [21], participants were exposed to the conversation in a more natural context; i.e. they were not informed that a conversation would take place; they only heard one conversation one time and, more importantly, were not aware that the conversation was part of the study. The current study’s realistic context allows it to be more generalizable to real-world situations in which people overhear a conversation. In addition, participants were asked to work on an attention task which they were told would impact their participation in the study, encouraging the participants to perform well on the task. By using a laboratory setting, we were able to control where the participant and confederates sat, the number of people in the room, and outside distractors. The laboratory setting also allowed us to test cognitive effects of overhearing the cell phone conversation.

In our experimental paradigm, participants were led to believe they were participating in a study examining the relationship between anagrams and reading comprehension. While the participant was completing an anagram task, the researcher left the room under the pretext of needing to make more copies. The participant overheard confederate(s) engaged in either a one-sided or two-sided conversation while the researcher was out of the room. After the researcher returned, she explained that the purpose of the study was to see how a conversation affected a person. The participant took a recognition test, assessing their memory for words from the conversation, and completed a questionnaire regarding the distractibility of the conversation. Based on abundant experimental and survey research showing the distracting nature of cell phones, we predicted one-sided conversations would be more distracting than two-sided conversations because of the unpredictable nature of a one-sided conversation. It was hypothesized that participants exposed to the one-sided conversation would be more distracted by it and would thus make more mistakes on the anagram task, but perform better on the memory task.

## Methods

### Ethics Statement

This study and consent process received approval from the University of San Diego’s Institutional Review Board. Participants provided their written consent to participate in this study. All participants were 18 years of age or older.

### Participants

Participants were 164 undergraduate students taking an Introduction to Psychology class at the University of San Diego. Fifteen participants guessed the purpose of the study and their data was not analyzed. Of the remaining 149 participants, 110 were female and 39 were male, with a mean age of 18.48 years (SD = .778, range = 18–21; one participant declined to list age). The ethnicities of the 149 participants were: 117 Caucasian, 15 Asian-American, 14 Hispanic, and 2 African American, and 1 Pacific Islander. Participants were recruited from the Psychology Department’s participant pool. Introductory Psychology students were required to either earn five research participation credits or perform alternative assignments. This study counted for two credits.

### Materials

#### Demographics

The demographics contained questions about age, gender, ethnicity, and primary spoken language. It also contained four questions about reading interests and word puzzles to maintain the perception that the study was about the relationship between anagram performance and reading comprehension.

#### Anagrams

Anagrams were used as an attention measure since the participant attempted to complete them while the conversation was taking place. Two types of scrambled words were used: *easy anagrams*, where first and last letters were in the correct position so that only middle letters are scrambled (e.g., hosue = house), and *hard anagrams*, where all of the letters were scrambled (e.g. suohe = house). This procedure was based on the methodology of Foley, Foley, Wilder, and Rusch [22]. The two levels of anagram difficulty have resulted in measureable differences in performance, such as in the time it takes to solve easy versus hard anagrams [23]. There were 15 easy anagrams and 15 hard anagrams. Words varied in length from 4–7 letters. The easy and hard categories had equal numbers of 4–7 letter words. In addition the average meaningfulness (easy = 6.66, hard = 6.61), imageability (easy = 6.18, hard = 6.36) and concreteness (easy = 6.08, hard = 6.46) were similar across the easy and hard categories according to the word ratings from Paivio, Yuille, and Madigan [24] and Fear [25].

#### Conversation

The conversation was a scripted conversation that lasted approximately seven minutes and covered three topics: a birthday party for dad, shopping for furniture, and meeting a date at the shopping mall. A one-sided conversation was conducted by a confederate talking on the cell phone; whereas two-sided conversations were carried out by two confederates speaking to each other. Confederates read the conversation off of a computer screen during the study. Each speaker said 506 words. Words that were tested on the recognition test only came from the conversation uttered by the speaker seated next to the participant. In both the one-sided and two-sided conversations, the confederate sitting next to the participant read from the same role in the script. None of the words on the recognition tests were repeated by the second speaker so that the participant only heard the recognition words one time, even if he/she overheard a two-sided conversation.

#### Manipulation Check

Participants answered the following three questions to determine if they knew the purpose of the study: “What do you think the purpose of this study is?”, “Do you have any questions about this study?” and “Do you think there is any other purpose to the study? If yes, please describe fully.”

#### Recognition Test

A recognition test was used to assess memory. This test took place approximately four minutes after the conversation ended. It consisted of seventy words from five categories. Participants were tested on their ability to discriminate (1) actual words from the conversation, (2) related words (same category as actual words), but not part of the conversation, and (3) other categories with varying relatedness to the conversation topic. Relatedness to the conversation topic was operationally defined as the number of words from that category that appeared in the conversation. For example, no words from the ‘beach’ category occurred during the conversation. In contrast, 10 words, such as “potatoes,” that fit into the food category appeared in the conversation. There were also six conversation words in the ‘school’ category and zero conversation words in the ‘household items’ category. None of these conversation food words appeared in the recognition test; for example “potatoes” did not appear in the recognition test, but a new word “salad” did. Mistakes were tracked to see if participants were more likely to make false alarms for food words as opposed to beach words. This procedure was meant to assess not only how many conversation words filter into the participant’s memory, but also what type of content was ‘picked up’ by the participant. During the recognition test, one word was shown on the computer screen at a time, and participants were asked to determine whether each word was part of the conversation they had just heard. To respond, the participant was asked to choose their confidence level on a scale of 1–6, from “definitely not” to “definitely yes.” Responses included: definitely not, probably not, maybe not, maybe yes, probably yes, and definitely yes.

#### Distractibility Scale

A seven item self-report questionnaire was administered on the distracting nature of the scripted conversation. The questionnaire was adapted from Monk et al. [19] and asked participants to rate how they felt while the conversation was taking place. It included the following statements: “I was surprised that the conversation was going on”, “The conversation was noticeable”, “I found myself listening to the conversation”, “Did you believe that the conversation was real?”, “The conversation was distracting”, “I found the volume of the conversation annoying”, and “I found the content of the conversation annoying.” The answers were on a seven point Likert scale which ranged from *strongly disagree* to *strongly agree*.

#### Post-Experiment Interview

At the end of the study, participants were asked about their thoughts regarding the study (“what were you thinking/feeling/considering doing?”), whether they were able to look at all the anagrams, and whether they were able to hear both sides of the one-sided conversation. They were also asked whether they had heard talk on campus about this particular study and were asked again about the purpose of the study.

### Procedure

Subjects were randomly assigned to one of three conditions:

1. two-sided conversation – participant heard both sides of a conversation taking place between two confederates.
2. one-sided/cell phone conversation – participant heard one side of a cell phone conversation taking place between a confederate and the researcher who was not in the room.
3. one-sided conversation with silent confederate – participant heard one side of a cell phone conversation, but there was also a second, silent, confederate present. This condition was used to determine if any differences between condition 1 and 2 were due to the different number of people in the room. It was thought that if the silent confederate appeared to ignore the conversation, the participant might also, making the results more similar to a two-sided conversation.

Participants were led to believe they were participating in a study examining the relationship between the ability to unscramble anagrams and reading comprehension. Trained confederates also appeared to be participating in the same study for research credits. For the two-sided conversation (Condition 1) and the one-sided conversation with silent confederate (Condition 3), two confederates showed up for the study; while one confederate showed up for the one-sided conversation (Condition 2). One participant was tested at a time.

The researcher indicated where each participant should sit. The room in which the experiment took place had three desks adjacent to each other, each with its own desktop computer. The desks were separated by a two-inch wide partial partition that extended approximately three feet from the wall and was five and a half feet high; the partition extended seven inches past the edge of the desks. The participant sat closest to the door and the confederates sat in the seats next to the participant, separated by the partition. The participant and the confederates always sat in the same seats so that the participant sat next to the confederate saying the half of the conversation that all participants would hear. This seating arrangement minimized differences between the volume of the words overheard that would be later tested on in the recognition task.

After seating the participant and confederate(s) and administering the informed consent, the researcher described the study as investigating the relationship between anagrams and reading comprehension. The researcher explained that the results of the anagram task would be used to determine which group each participant would be placed in for the reading comprehension portion of the study, thus imparting some consequences on the participant’s anagram performance. The researcher gave the participant a copy of the anagrams to unscramble. The researcher then pretended to realize that the other anagram copies were bad copies in which half the anagrams were not printed. The researcher showed the participant and confederate(s) the bad copies, apologized, and explained that he/she needed to leave the room to make more copies of the anagrams for the other subject(s) (the confederate(s)). The researcher instructed the participant to go ahead and complete the anagrams since they were only part of the ‘pre-experiment’, and that the researcher would pick up the participant’s anagram answer sheet upon the researcher’s return. After the researcher left the room, the confederate either received a phone call from the researcher or engaged in a conversation with the other confederate. For one-sided conversations, the cell phone was placed out of view on the desk and was answered after the first vibration. During the one-sided conversations, the confederate sat next to the participant and read from the script. For the two-sided conversations, only the confederate sitting next to the participant said the words that were later included in the recognition task.

Upon the researcher’s return, the participant was given the manipulation check in which they were asked to write down what they thought was the purpose of the study. The confederates were then excused and the participant was given a surprise recognition memory test on the computer using MediaLab (Empirisoft). Finally, the participant was asked a series of questions regarding the distracting nature of the conversation (whether it was loud, annoying, believable, etc.) on a Likert scale of 1–7, and were interviewed about their thoughts regarding the study. The participant was then debriefed, asked to maintain confidentiality regarding the study, and thanked for their time.

## Results

Participants’ data were not analyzed if the participant guessed the purpose of the study (n = 15). This left 60 participants who overheard the two-sided conversation, 56 who overheard the one-sided conversation, and 33 participants who overheard the one-sided conversation with a silent confederate. A three-way analysis of variance (ANOVA) showed that the one-sided and one-sided with silent confederate conditions were not significantly different from each other on either the attention or memory tests (see Recognition test and d’) so these two conditions were combined, leaving 60 participants who overheard the two-sided conversation, and 89 who overheard the one-sided conversation. A Chi-square test comparing participants in the three conditions revealed that participants in the three conditions were not significantly different in regards to gender, ethnicity, and primary spoken language (Table 1) or age (Table 2). A second Chi-square test comparing participants in the combined one-sided group versus the two-sided group also showed that participants were not significantly different in their demographic characteristics (Tables 3 and 4).

**Table 1:** Demographic information for participants in the one-sided, two-sided, and one-sided with silent confederate conditions.

		Conversation Type					
		one-sided		two-sided		one-sided silent	
		N	%	N	%	N	%
Gender	Female	45	80.4	43	71.7	22	66.7
	Male	11	19.6	17	28.3	11	33.3
Ethnicity	White	46	82.1	47	78.3	24	72.7
	Hispanic	4	7.1	6	10.0	4	12.1
	Asian	5	8.9	6	10.0	4	12.1
	Pacific Islander	1	1.8	0	0.0	0	0.0
	African American	0	0.0	1	1.7	1	3.0
First Language	English	52	92.9	54	9.0	29	87.9
	Other	4	7.1	6	1.0	4	12.1

**Table 2:** Average age for participants in the one-sided, two-sided, and one-sided with silent confederate conditions.

	Conversation Type		
	one-sided	two-sided	one-sided silent
	µ (SD)	µ (SD)	µ (SD)
Age	18.4 (0.71)	18.5 (0.79)	18.6 (0.87)

**Table 3:** Demographic information for participants in the one-sided and two-sided conditions.

		Conversation Type			
		one-sided		two-sided	
		N	%	N	%
Gender	Female	67	75.3	43	71.7
	Male	22	24.7	17	28.3
Ethnicity	White	70	78.7	47	78.3
	Hispanic	8	9.0	6	10.0
	Asian	9	12.5	6	10.0
	Pacific Islander	1	1.4	0	0.0
	African American	1	1.4	1	1.7
First Language	English	81	91.0	54	9.0
	Other	8	9.0	6	1.0

**Table 4:** Average age for participants in the one-sided and two-sided conditions.

	Conversation Type	
	one-sided	two-sided
	µ (SD)	µ (SD)
Age	18.5 (0.77)	18.5 (.79)

### Distractibility Scale

A Cronbach’s alpha was used to determine whether the seven statements of the distractibility scale were measuring the same concept. The distractibility scale had a Cronbach’s alpha of.752. The item measuring the believability of the conversation had a corrected item-total correlation of.072. Since the believability was not related to the distractibility of the conversation, it was dropped from further distractibility analysis. Believability was significantly different between groups (one-sided, M = 6.31, SD = 1.21; two-sided, M = 5.71, SD = 1.93; F(1) = 5.102; p = .025. However, further analyses revealed that believability was not related to performance on either the anagram or recognition task, or to any performance differences between the one-sided and two-sided conditions. The Cronbach’s alpha for the remaining six questions on the distractibility scale was.796.

An overall distractibility score was calculated for each participant by summing each participant’s responses to the remaining six items of the distractibility scale. An independent sample t-test indicated participants exposed to the one-sided conversation reported more distraction (M = 28.517, SD = 5.872) than those exposed to the two-sided conversation (M = 22.700, SD = 7.797; t(102.682) = 4.915, p<.001). A comparison between conditions indicated that the distractibility scale did not meet Levene’s test of equality of variances (F = 5.566 and p = .020) and therefore, we adjusted the degrees of freedom from 147 to 102.682.

**Figure 1:**
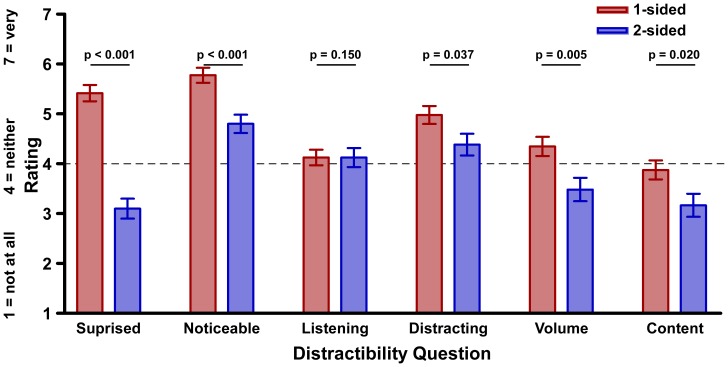
Average responses to distractibility scale. Participants’ averaged responses to statements on the distractibility scale. For example, responses to the statement “While the conversation was taking place, the conversation was noticeable” could range from 1, “not at all noticeable,” to 7, “very noticeable.” A response of 4 indicated “neither noticeable nor not noticeable.”

A multivariate analysis of variance (MANOVA) compared the responses between conditions to the individual questions on the distractibility scale (Figure 1). Participants who overheard the one-sided conversation were more surprised that the conversation took place (p<.0001), and rated the conversation as more noticeable (p<.0001), and distracting (p = .037) than those who overheard the two-sided conversation. They were also more likely to rate the content (p = .020) and volume (p = .005) of the conversation as annoying.

### Anagram Data

**Figure 2:**
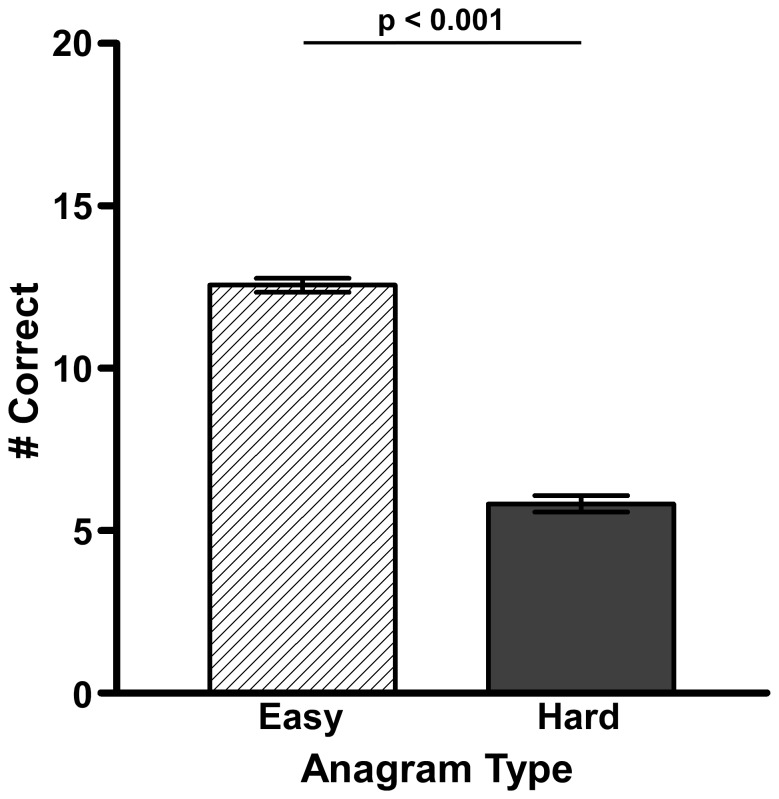
Average number of correct easy and hard anagrams. The average number of easy and hard anagrams that each participant completed correctly, regardless of which conversation they overheard.

**Figure 3:**
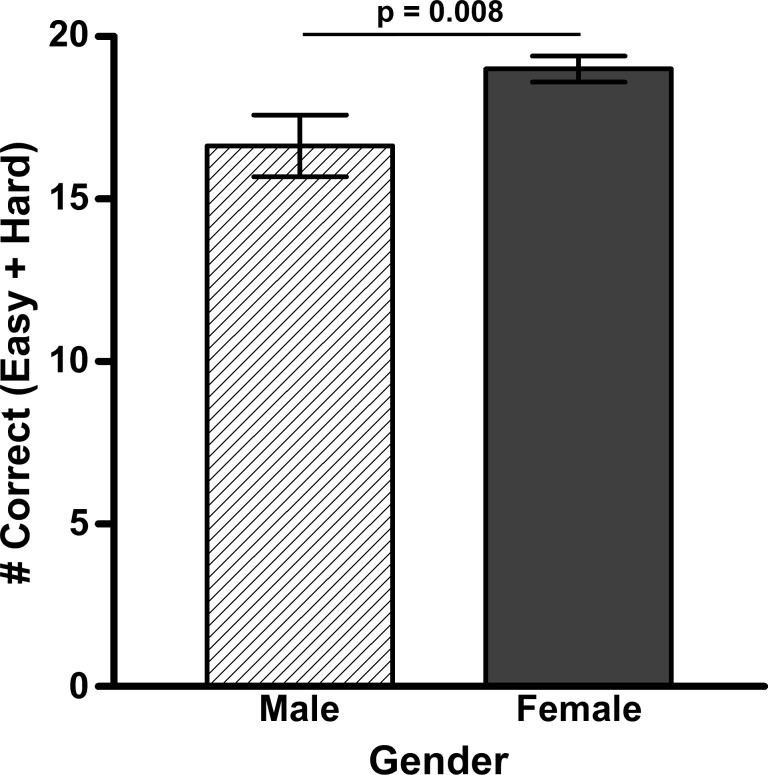
Average number of correct and incorrect anagram responses by gender. The average number of easy and hard anagrams that males and females answered correctly, regardless of which conversation they overheard.

The number of anagrams that were solved correctly and incorrectly was tallied. Incorrect anagrams included responses in which participants crossed out or erased their answers as well as wrong answers. Paired-samples t-test revealed that participants correctly solved more easy anagrams (M = 12.557, SD = 2.613) than hard anagrams (M = 5.826, SD = 3.106; t(148) = 26.150, p<.001; Figure 2). There was a significant difference between the total number of anagrams that females (M = 19.000, SD: 4.195) and males (M = 16.641, SD: 5.932) correctly solved; t(52.10) = −2.289, p = .026 (Figure 3). The number of anagrams solved correctly did not meet Levene’s test of equality of variances (F = 6.854 and p = .01) and therefore, we adjusted the degrees of freedom from 147 to 52.10. However, there were no significant differences between the two-sided and one-sided conversations in regards to number of anagrams solved correctly or incorrectly. In addition, the number of easy versus hard anagrams that were solved correctly also did not vary by condition (one-sided easy/hard anagrams: 12.43/6.11; two-sided easy/hard: 12.75/5.4) as revealed by a 2×2 ANOVA (anagram type×conversation type).

### Recognition Test and d’

For the recognition task, participants’ responses were measured in terms of d’, a formula that takes into account each participant’s ability to correctly discriminate stimuli amongst novel distractors [26]. d’ takes into account “hits” (correct identifications of repeated words), “misses” (failures to identify repeated words), “correct rejections” (correct identifications of novel words), and “false alarms” (mistakenly identifying a novel word as a repeated word). The formula is *d'* = *Z*(hit rate) − *Z*(false alarm rate). Hit rate = hits/(hits+misses). False alarm rate = false alarms/(false alarms+correct rejections). In cases where the response rate was 0, the response was converted to 1/(2N) where *N* = the number of trials [27].

A three way ANOVA comparison of the d’ for the on-sided condition versus the two-sided condition and one-sided condition with silent confederate revealed a main effect for conversation type. (one-sided conversation, M = .65, SD = .56; two-sided conversation, M = .31 SD = .48; and one-sided with silent confederate, M = .57, SD = .69; F(2) = 5.487, p = .005). Post hoc tests revealed that one-sided condition vs. one-sided with silent confederate condition were not significantly different from each other, p = .521. However, the one-sided condition was significantly different from the two-sided condition, p = .002, and the one-sided condition with silent confederate was also significantly different from the two sided condition, p = .038. Since the one-sided condition and the one-sided condition with silent confederate were not significantly different from each other but both of these groups were significantly different from the two-sided group, we collapsed the data for the one-sided group with the one-sided with silent confederate group.

**Figure 4:**
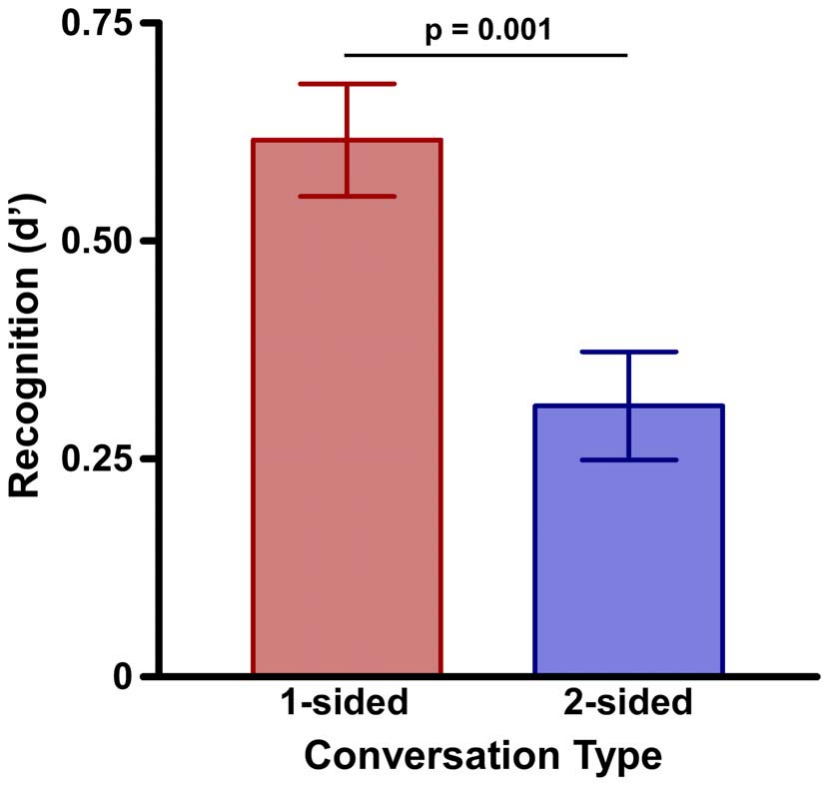
Average d’ for each conversation type. The average d’ score for participants who overheard a one-sided versus two-sided conversation.

There was a significant effect of conversation type on d’ (Figure 4). An independent samples t-test revealed that participants exposed to a one-sided conversation had a larger d’ score on the recognition test (M = .616, SD = .608) than those who overheard a two-sided conversation (M = .311, SD = .481); t(147) = 3.256, p = .001. We initially restricted data analysis to only those participants who rated the conversation as moderately or highly believable (believability > = 4, then believability > = 5, etc), but the significance values on the recognition test did not change to any notable degree. A univariate ANOVA revealed no interaction between gender and conversation type. We analyzed the individual components that are factored into d’ and found no significant differences between conversation types on false alarms made to the five word categories on the recognition task. However, participants who overheard the one-sided conversation correctly identified more words from the conversation, i.e. made more “hits”, (M = 4.47, SD = 2.216) than those who overheard the two-sided conversation (M = 3.75, SD = 1.819); t(147) = 2.092, p = .038.

### Recognition Test and Confidence Ratings

**Figure 5:**
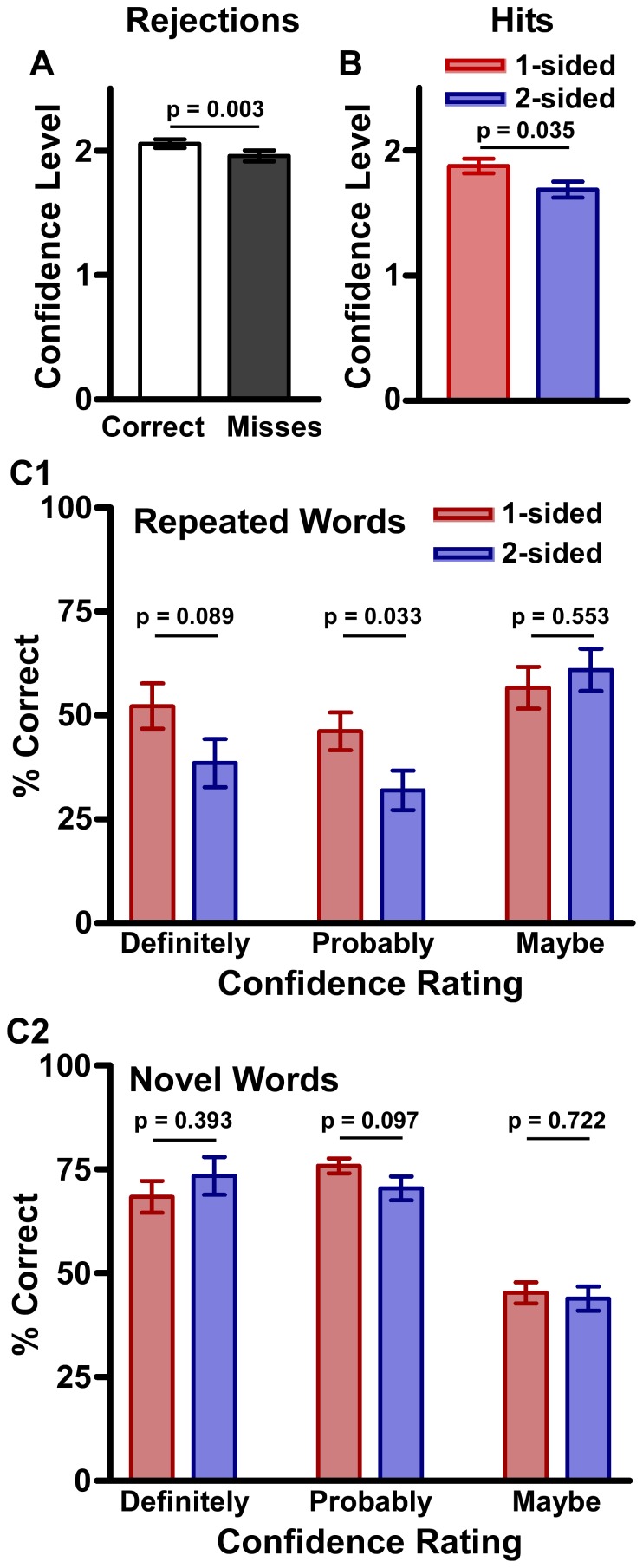
Recognition Test and Confidence Ratings. 5A. Confidence levels for correct rejections and misses, regardless of which conversation participants overheard. 5B. Confidence levels for hits for participants who overheard a one-sided versus two-sided conversation. 5C. The accuracy of different types of confidence ratings (definitely, probably, maybe) on the recognition test. Responses are shown for participants who overheard one-sided versus two-sided conversations. 5C1. Accuracy of responses to repeated words. 5C2. Accuracy of responses to novel words.

The confidence levels for different types of responses were compared. Participants were more confident in their correct rejections (M = 2.058, SD = .428) than their misses (M = 1.960, SD = .545; paired samples t-test; t(148) = 2.993; p = .003; Figure 5A), but there were no significant differences between one-sided and two-sided conditions in regards to correct rejections or misses. In contrast, participants who overheard the one-sided conversation were more significantly more confidant in their ‘hits’ (M = 1.877, SD = .545) than participants who overheard the two-sided conversation (M = 1.687, SD = .495; t(142) = 2.13, p = .035; Figure 5B).

The accuracy for each type of confidence rating was also calculated: 1) definitely (definitely yes and definitely not), 2) probably (probably yes and probably not), and 3) maybe (maybe yes and maybe not)(Figure 5C1 and 5C2). A multivariate anova (MANOVA) revealed a significant difference for accuracy of “probably” ratings of words from the conversation (one-sided conversation: M = 46.18, SD = 33.94; two-sided conversation: M = 31.96, SD = 32.14; F(1) = 4.649, p = .033).

## Discussion

We hypothesized that the self-report questionnaire, anagram task, and recognition task would reveal that participants who overheard the one-sided conversation would be more distracted than participants who overheard the two-sided conversation. As predicted, participants exposed to the one-sided conversation did report being more distracted by the conversation than those who overheard the two-sided conversation. In addition, those who overheard the one-sided conversation had a more accurate performance on the recognition task, and were more confident and accurate in their responses to words from the conversation. Although participants completed more easy than hard anagrams, and females performed better on the anagram task than males, there were no significant differences between the different conversation conditions on the anagram task.

Several components of the distractibility questionnaire were significantly different between the two conditions. Participants who overheard the one-sided conversation rated the conversation as more noticeable, and distracting. They were also more surprised that the conversation took place than participants in the two-sided condition. Participants who overheard the one-sided conversation were also more likely to rate the content and volume of the conversation as annoying than those who overheard the two-sided conversation. These results are in agreement with those of Monk et al. [19], [20]. Their participants rated the one-sided conversation as more noticeable and intrusive, and were more likely to find themselves listening to it. One important note is that Monk’s participants found the volume more annoying even though the researchers controlled for volume level of the conversation [19].

The annoyance that participants who overheard the one-sided conversation felt is consistent with surveys that have shown people are annoyed by other’s cell phone use in public [5], [18]. This annoyance may be caused by the “blurring of the distinction between the public and the private sphere” [28]. For example, people typically have personal, not business, conversations while they use cell phones in public [2]. Bystanders who are exposed to these personal conversations may not have much control over the situation, thereby increasing their levels of annoyance and frustration. Research has shown that bystanders in situations where they are not free to leave (for example, waiting for or using public transportation) often find cell phone conversations annoying [18], [19]. Other research investigating the effects of lack of control have shown that lack of perceived control can, in turn, lead to an increase in stress responses [29], [30].

In regards to results of the anagram task, females have been shown to perform better than males on anagrams tasks [31], and it’s not surprising that participants would complete more easy than hard anagrams. However, we did expect to see a difference in anagram performance between conditions and did not. Emberson et al. [21] observed significant differences between the silent condition and one-sided conversation, but not between the two-sided conversation and silence, on their choice reaction and motor tracking tasks. It is interesting to note that Emberson et al. did not report any differences across attentional measures (verbal or motor) between their one-sided and two-sided groups. Since previous research did not reveal differences between conversation types on their primary attention tasks, we concluded that perhaps a different task that allows more attentional resources to be paid to distractors might reveal attentional differences. Lavie [32] has suggested, with support from many studies [33], that distractors have a larger impact on an easy task versus a harder task. The reason is that less attentional resources are used in the easy task, leaving participants free to be distracted by irrelevant stimuli. Not only does task load affect performance on the primary task, but it also affects memory for the irrelevant distractors [34]. Memory for unattended distractors is greater on tasks with low loads. We had equal number of easy and hard anagrams. In the easy anagrams, the first and last letters were in the correct position so that the middle letters were scrambled; in the hard anagrams, all of the letters were scrambled. Perhaps a future study using a greater number of easy anagrams would reveal differences between conditions on our paradigm.

In this study, participants who overheard the one-sided conversation performed better on the recognition memory task than those who overheard the two-sided conversation, indicating that they remembered more words that were said in the conversation. The one-sided condition’s better memory for the conversation occurred despite an experimental design in which participants were not asked to focus on the stimuli of interest. In memory studies, the researcher typically knows that the participants attended to the stimuli; for example, the participant is asked to study the stimuli or there is only one set of stimuli (e.g., [35]). Even in covert memory studies, participants are often asked to rate the stimulus on a particular dimension (e.g., [36]). However, in this study, whether or not participants paid attention to the stimulus was a decision that was left to each participant. Many participants said that they tried to ignore the stimulus. This type of design likely minimized differences between the two conversation conditions. Thus, our paradigm may have attenuated overall memory performance, yet exposed unique, intrusive, "attention grabbing" aspects of a one-sided cell phone conversation.

Many studies have examined performance regarding unattended stimuli (e.g., [37], [38], [39]). The responses to the unattended conversation in this study were stronger than those in some studies [34], [39], [40]. However, it is difficult to compare our results with these studies since their stimuli were very brief, 170 to 500 ms, and participants were instructed to attend to the target stimuli and ignore distractors. Another factor that may have improved performance in our study is that the stimuli were in two different modalities, visually-presented anagrams and auditory distractors. According to multiple resources theory [41], performance on tasks with simultaneously-presented stimuli from multiple modalities shows less decrement than competing stimuli from the same modality. This occurs because the multimodal stimuli are using different attentional resources, and thus are not competing for the same resources. It could be that the visually-presented anagrams did not use the same attentional resources as the conversation and thus made it easier for participants to process the conversation. Perhaps the primary reason we found differences in d’ performance is that participants chose to attend to the conversation despite the researcher telling them that their performance on the anagram task would impact their participation in the rest of the study and that their answer sheet would be picked up upon the researcher’s return. Lavie notes that this may also be a factor in studies which do explicitly ask their participants to ignore irrelevant stimuli: “According to the load model, the allocation of attention is an automatic process in the sense that it cannot be simply withheld at will because of the instruction to ignore task-irrelevant objects” [34]. In our study, every participant attempted the anagrams, and many participants appeared to pay some attention to the conversation during the anagram task based on their performance on the memory task. This study adds to the body of research by suggesting that overhearing a cell phone conversation competes for attentional resources that may have otherwise been devoted to other tasks.

There were also significant differences between conditions in the accuracy of high confidence responses on the recognition test. Participants in the one-sided condition were more likely to be correct when they said that words were “probably” part of (or not part of) the conversation. When participants were unsure about a word’s presence in the conversation and chose “maybe”, there were no significant differences between groups. Hoffman et al. [38] have suggested that memory studies may have missed the retention of unattended stimuli. They argue that the retention of unattended stimuli is detectable if the confidence ratings for “no” responses (i.e., the participant says the word is not a repeated word) are studied. They found that “no” responses to unattended stimuli (misses) were less confident compared to “no” responses to novel stimuli (correct rejections). Our comparison of confidence levels for misses and correct rejections (mistakes versus correct responses) revealed results similar to Hoffman et al.; participants were more confident in their correct rejections than their misses. In addition, participants who overheard the one-sided conversation were more confident in their ‘hits’ and their moderately high (‘probably’) confidence responses were more accurate than those in the two-sided condition. The stronger confidence ratings and accuracy of the one-sided group could be due to some of the factors that may have improved d’ performance in our study: longer stimuli, different stimuli modalities, and participants choosing to pay attention to the conversation.

The main strength of the current study is its realistic design. Participants were led to believe that the conversation was not part of the study. Similar to cell phone conversations in natural settings, the conversations in our study had an element of surprise and bystanders to the conversation decided themselves whether or not to attend to the conversation. When people are distracted by a conversation while working on a task, they are not usually warned a conversation will take place. Moreover, if participants had been asked to ignore the conversation, it might lessen the generalizability of the study. The current study is also unique in several other regards. First, the participants heard the conversation only one time as people would in naturalistic settings. Second, participants were tested on two cognitive abilities, attention to the anagram task during the conversation and memory for the conversation after it ended.

A possible limitation of this study may be the difference in number of words overheard in the two types of conversations. Compared to the two-sided conversation, those who overheard the one-sided conversation heard only half of the words. It’s possible that participants who overheard the one-sided conversation performed better on the recognition task because of experimental confounds. Participants in the one-sided condition heard less noise and had less information to remember, and possibly the confederates spoke louder during the one-sided conversation than the two-sided conversation. We did not control for the difference in sound and word count which may have contributed to the differences in distraction. Nevertheless, in Emberson et al. [21], the participants in the one-sided conversation performed worse than those in the two-sided conversation when both groups were compared to the silent condition. The poorer performance of their one-sided group occurred even though this group was exposed to 42% less words and noise. They attributed the poor performance of the one-sided group to the unpredictability of the one-sided conversation’s content, and not the difference in amount of speech overheard. Emberson et al.’s [21] hypothesis was supported by results in a follow-up study in which performance did not differ between groups when conversation content was filtered out, even though the two-sided group was exposed to almost twice as much noise. Compared to predictable noises, unpredictable noises appear to be better distractors because they caused slower reaction times. However, when participants were given instructions to pay attention to the sounds, all participants had slower reaction times. Participants were able to tune out the predictable better than they could random sounds [42]. In our study, the one-sided cell phone conversation, with the missing content, could be described as unpredictable. This supports previous research findings that one-sided conversations are less predictable because they are missing content [21].

Although the volume for each conversation type was not recorded, attempts were made to ensure that each participant was tested under similar conditions. For example, in order to reduce between-subject volume differences, only the confederate sitting next to the participant said the words that were tested on the recognition task. While participants in the one-sided conversation were more likely to rate the volume as annoying, the average rating for the one-sided conversation was 4.35 on a scale of 1 to 7, midway between a score of 4, neither distracting nor not distracting and 5, slightly distracting. Thus it seems that the volume of the one-sided conversation was only mildly annoying. It’s possible that participants rated the volume of the one-sided conversation as more annoying even though there were no volume differences between the conversations. Prior research has shown that participants can rate the volume of one-sided conversations as more annoying even when volume is controlled [19].

Although, we did not record volume levels for the conversations described in this study, we have completed a follow-up study in which volume was recorded for 54 participants (n = 27 one-sided; 27 two-sided). The conversation in the follow-up study was not identical to the one described in this paper, but was centered on the same list of words and was thus tested with the same recognition test. Although, there were no significant differences in volume between the two groups (one-sided volume = 55.09dB; two-sided volume = 54.38dB), the d’ for the 1 one-sided conversation was significantly greater than the d’ for the two sided conversation (p = .028). Since both studies were conducted using the same procedure in the same room, and d’ was significantly different despite equal volume levels in the follow-up study, we have reason to think volume probably played a negligible role in this study.

It is likely that cell phone conversations would impact a bystander’s cognition. Monk and colleagues [20] theorize that people have a tendency to want to complete information that is missing in order to make it understandable. Similarly, Hughes, Vachon, and Jones [43] believe that people attempt to process and integrate peripheral information (even if it’s to be ignored), and when it is not possible to process and integrate, attention is captured. Thus, it is expected that a one-sided cellphone conversation would negatively impact a bystander’s cognitive processing. Emberson and colleagues [21] demonstrated that unpredictable content, but not unpredictable noise, negatively affect performance. They also explored whether errors were due to participants’ attempts to fill in the missing conversation during the silent periods of the one-sided conversation. Participants made more errors when the one-sided speaker began speaking after a period of silence, in comparison with periods of silence and two-sided conversations. Emberson et al [21] reason that bystanders to cell phone conversation, unlike those exposed to dialogues, do not know what turn the conversation will take when the cellphone user speaks again after a silence. Attempting to process the conversation without the context provided by the prior speaker increases the cognitive processing that a bystander must use.

This study is the first to have observed cognitive effects of cell phone conversations on bystanders in a realistic context. Participants rated the cell phone conversation as more distracting and were more likely to remember content from the conversation, even though they were working on another task and were unaware that the conversation was part of the study. These results have implications for workplace environments, transportation hubs and other public areas. Future studies should explore how attention and cognitive effects of cell phone use vary as a function of conversation volume and content. Additionally, it will be important to determine what types of tasks are subject to performance impairments by overheard cell phone conversations.
